# A mixed integer linear programming model to reconstruct phylogenies from single nucleotide polymorphism haplotypes under the maximum parsimony criterion

**DOI:** 10.1186/1748-7188-8-3

**Published:** 2013-01-23

**Authors:** Daniele Catanzaro, Ramamoorthi Ravi, Russell Schwartz

**Affiliations:** 1 Graphes et Optimisation Mathématique (G.O.M.), Computer Science Department, Université Libre de Bruxelles (U.L.B.), Boulevard du Triomphe, CP 210/01, B-1050, Brussels, Belgium; 2 Tepper School of Business, Carnegie Mellon University, 5000 Forbes Avenue, Pittsburgh, PA 15213-3890; 3 Department of Biological Sciences and Lane Center for Computational Biology, Carnegie Mellon University, 4400 Fifth Avenue, Pittsburgh, PA, 15213

**Keywords:** Combinatorial optimization, Exact algorithms, Mixed integer programming, Phylogeny estimation, Haplotype estimation, Maximum parsimony, Single nucleotide polymorphism, Computational biology

## Abstract

**Background:**

Phylogeny estimation from aligned haplotype sequences has attracted more and more attention in the recent years due to its importance in analysis of many fine-scale genetic data. Its application fields range from medical research, to drug discovery, to epidemiology, to population dynamics. The literature on molecular phylogenetics proposes a number of criteria for selecting a phylogeny from among plausible alternatives. Usually, such criteria can be expressed by means of objective functions, and the phylogenies that optimize them are referred to as optimal. One of the most important estimation criteria is the *parsimony* which states that the optimal phylogeny *T*^∗^for a set H of *n* haplotype sequences over a common set of variable loci is the one that satisfies the following requirements: (i) it has the shortest length and (ii) it is such that, for each pair of distinct haplotypes $h_{i},h_{j}\in H$, the sum of the edge weights belonging to the path from *h*_*i*_ to *h*_*j*_ in *T*^∗^ is not smaller than the observed number of changes between *h*_*i*_ and *h*_*j*_. Finding the most parsimonious phylogeny for H involves solving an optimization problem, called the *Most Parsimonious Phylogeny Estimation Problem* (MPPEP), which is NP-hard in many of its versions.

**Results:**

In this article we investigate a recent version of the MPPEP that arises when input data consist of single nucleotide polymorphism haplotypes extracted from a population of individuals on a common genomic region. Specifically, we explore the prospects for improving on the implicit enumeration strategy of implicit enumeration strategy used in previous work using a novel problem formulation and a series of strengthening valid inequalities and preliminary symmetry breaking constraints to more precisely bound the solution space and accelerate implicit enumeration of possible optimal phylogenies. We present the basic formulation and then introduce a series of provable valid constraints to reduce the solution space. We then prove that these constraints can often lead to significant reductions in the gap between the optimal solution and its non-integral linear programming bound relative to the prior art as well as often substantially faster processing of moderately hard problem instances.

**Conclusion:**

We provide an indication of the conditions under which such an optimal enumeration approach is likely to be feasible, suggesting that these strategies are usable for relatively large numbers of taxa, although with stricter limits on numbers of variable sites. The work thus provides methodology suitable for provably optimal solution of some harder instances that resist all prior approaches.

## Background

Molecular phylogenetics studies the hierarchical evolutionary relationships among species, or *taxa*, by means of molecular data such as DNA, RNA, amino acid or codon sequences. These relationships are usually described through a weighted tree, called a *phylogeny*, whose *leaves* represent the observed taxa, *internal vertices* represent the intermediate ancestors, *edges* represent the estimated evolutionary relationships, and *edge weights* represent measures of the similarity between pairs of taxa.

Accurately characterizing evolutionary relationships between organisms and their genomes is the basis of comparative genomic methods for interpreting meaning in sequence data, and for this reason the use of molecular phylogenetics has become widely used (and sometimes indispensable) in a multitude of research fields such as systematics, medical research, drug discovery, epidemiology, and population dynamics [3]. For example, the use of molecular phylogenetics was of considerable assistance in predicting the evolution of human influenza A [4], understanding the relationships between the virulence and the genetic evolution of HIV [5,6], identifying emerging viruses as SARS [7], recreating and investigating ancestral proteins [8], designing neuropeptides causing smooth muscle contraction [9], and relating geographic patterns to macroevolutionary processes [10].

The literature on molecular phylogenetics proposes a number of criteria for selecting the phylogeny of a set H of haplotypes extracted from *n* taxa from among plausible alternatives. The criteria can usually be quantified and expressed in terms of objective functions, giving rise to families of optimization problems whose general paradigm can be stated as follows [11]:

### Problem 1

– *The Phylogeny Estimation Problem (PEP)*

$$\begin{array}{ccc} \text{optimize} & f(T)\\ \;\;\mathrm{s.t.} & g(H,T)=1\\ & T\in T, & \end{array}$$

where *T* a phylogeny of H,T the set of all possible phylogenies of H,f:T→R a function modeling the selected criterion of phylogeny estimation, and g:H×T→R is a characteristic function equal to one if a phylogeny *T* is compatible (according to the selected criterion) for the set H. A specific optimization problem is completely characterized by defining the functions *f* and *g*, and the phylogeny *T*^∗^ that optimizes *f* and satisfies *g* is referred to as *optimal*.

One of the classic criteria for phylogeny estimation is the *parsimony criterion*, which assumes that one taxon evolves from another by means of small changes and that the most plausible phylogeny will be that requiring the smallest number of changes. That evolution proceeds by small rather than smallest changes is due to the fact that the neighborhood of possible alleles that are selected at each instant of the life of a taxon is finite and, perhaps more important, that the selective forces acting on the taxon may not be constant throughout its evolution [12,13]. Over the long term (periods of environmental change, including the intracellular environment), the accumulation of small changes will not generally correspond to the smallest possible set of changes consistent with the observed final sequences. Nevertheless, it is plausible to believe, at least for well-conserved molecular regions where mutations are reasonably rare and unlikely to have occurred repeatedly at any given variant locus, that the process of approximating small changes with smallest changes could provide a good approximation to the true evolutionary process of the observed set of taxa [14]. Such an assumption is likely to be reasonable, for example, in intraspecies phylogenetics, where few generations have elapsed since the observed taxa shared a common ancestor and thus the expected number of mutations per locus is much less than one. When such assumtions hold, a phylogeny of H is defined to be optimal under the parsimony criterion if it satisfies the following requirements: (i) it has the shortest length, i.e., the minimum sum of the edge weights, and (ii) it is such that, for each pair of distinct haplotypes $h_{i},h_{j}\in H,$ the sum of the edge weights belonging to the path from *h*_*i*_ to *h*_*j*_ in *T*^∗^ is not smaller than the observed number of changes between *h*_*i*_ and *h*_*j*_[11]. The first condition imposes the assumption that the smallest number of mutations consistent with the observed sequences is a good approximation to the true accumulated set of mutations; the second condition correlates the edge weights to the observed data.

The parsimony assumption can be considered accurate in the limit of low mutation rates or short time scales, making it a reasonable model for situations such as analysis of intraspecies variation where little time is presumed to have elapsed since the existence of a common ancestor of all observed taxa. Maximum parsimony also remains valuable as a model for novel methodology development in phylogenetics because of its relative simplicity and amenity to theoretical analysis. Novel computational strategies, such as those developed in this paper, might therefore productively be developed and analyzed in the context of maximum parsimony before being extended to more complicated models of phylogenetics.

Finding the phylogeny that satisfies the parsimony criterion involves solving a specific version of the PEP, called the *Most Parsimonious Phylogeny Estimation Problem* (MPPEP). Some of the variants of the MPPEP, see e.g., [15,16], can be solved in polynomial time, however, in the most general case, the problem is NP-hard [11,17] and this fact has justified the development of a number of exact and approximate solution approaches, such those described in [11,17,18]. Some recent versions of the MPPEP, such as the Most Parsimonious Phylogeny Estimation Problem from SNP haplotypes (MPPEP-SNP) investigated in this article, play a fundamental role in providing predictions of practical use in several medical bioinformatics applications, such as disease association studies [19] or reconstruction of tumor phylogenies [20,21]. In these contexts, it would be highly desirable to have the most accurate inferences possible for specific applications, but this in turn would imply to have algorithms able to exactly solve instances of such versions. As regards the MPPEP-SNP, the literature describes some (rare) circumstances for which it is possible to solve the problem in polynomial time (see Section Methods). Unfortunately, in the general case the MPPEP-SNP is NP-hard and solving provably to optimality therefore generally requires the use of exact approaches based on implicit enumeration algorithms, similar to the mixed integer programming strategies described in [1,2,22].

In this article, we explore the prospects for improving on the implicit enumeration strategy of [1,2] using a novel problem formulation and a series of additional constraints to more precisely bound the solution space and accelerate implicit enumeration of possible optimal phylogenies. We present a formulation for the problem based on an adaptation of [23]’s mixed integer formulation for the Steiner tree problem extended with a number of preprocessing techniques and reduction rules to further decrease its size. We then show that it is possible to exploit the high symmetry inherent in most instances of the problem, through a series of strengthening valid inequalities, to eliminate redundant solutions and reduce the practical search space. We demonstrate through a series of empirical tests on real and artificial data that these novel insights into the symmetry of the problem often leads to significant reductions in the gap between the optimal solution and its non-integral linear programming bound relative to the prior art as well as often substantially faster processing of moderately hard problem instances. More generally, the work provides an indication of the conditions under which such an optimal enumeration approach is likely to be feasible, suggesting that these strategies are usable for relatively large numbers of taxa, although with stricter limits on numbers of variable sites. The work thus provides methodology suitable for provably optimal solution of some harder instances that resist all prior approaches. In future work, it may provide useful guidance for strategies and prospects of similar optimization methods for other variants of phylogeny inference.

## Methods

### Notation and problem formulation

Before proceeding, we shall introduce some notation and definitions that will prove useful throughout the article. The human genome is divided in 23 pairs of chromosomes, i.e., organized structures of DNA that contain many genes, regulatory elements and other nucleotide sequences. When a nucleotide site of a specific chromosome region shows a variability within a population of individuals then it is called a *Single Nucleotide Polymorphism* (SNP). Specifically, a site is considered a SNP if for a minority of the population a certain nucleotide is observed (called the minor allele) while for the rest of the population a different nucleotide is observed (the major allele). The minor allele, or *mutant type*[24], is generally encoded as ‘1’, as opposed to the major allele, or *wild type*[24], generally encoded as ‘0’. A haplotype is a set of alleles, or more formally, a string of length *m* over an alphabet Σ = {0,1} [25].

Given a set H of *n* haplotypes, denote S={1,2,…,m} as the set of alleles and *h*_*i*_(*s*), s∈S, as the *s*-th allele of haplotype $h_{i}\in H$. Given two distinct haplotypes $h_{i},h_{j}\in H,$ we denote $S_{h_{i}h_{j}}$ as the subset of different alleles between *h*_*i*_ and *h*_*j*_, $d_{h_{i}h_{j}}=\underset{s\in S_{h_{i}h_{j}}}{\sum }|h_{i}(s)-h_{j}(s)|$ as the *distance* between *h*_*i*_ and *h*_*j*_, and we say that *h*_*i*_ and *h*_*j*_ are *adjacent* if $d_{h_{i}h_{j}}=1$. From a biological point of view, the adjacency between a pair of distinct haplotypes means that one of the two haplotypes evolved from the other by mutation of a specific SNP over time.

Consider a graph G=(H,E) having a vertex for each haplotype in H and an edge for each pair of adjacent haplotypes $h_{i},h_{j}\in \text{H.}$ Then, a *phylogeny T* of H is a spanning tree of *G*, i.e., an acyclic subgraph of *G* in which a pair of vertices $h_{i},h_{j}\in H$ is adjacent in *T* if $d_{h_{i}h_{j}}=1$. It is worth noting that, according to the definition of the edge set *E*, in general a phylogeny of H may not exist as the graph G=(H,E) might not be connected. To always ensure the existence of a phylogeny for H, we introduce the set $H^{\prime }$ which consists of the minimum number of haplotypes that should be added to H in such a way that, defined $\bar{H}=H\cup H^{\prime }$ and $\bar{E}=\{(h_{i},h_{j}):h_{i},h_{j}\in \bar{H}\;\text{and}\;d_{h_{i}h_{j}}=1\}$, the graph $\bar{G}=(\bar{H},\bar{E})$ is connected. From a biological point of view, the set $H^{\prime }$ represents the set of haplotypes that are ancestors of the observed ones but either had gone extinct or just were not observed in that sample (also called Steiner nodes).

Denote $\bar{T}$ as a phylogeny of $\bar{H}$, $\bar{E}(\bar{T})$ as the edge set of $\bar{T}$, and $L(\bar{T})$ as the *length* of the phylogeny $\bar{T}$, i.e., the sum of the distances $d_{h_{i}h_{j}}$, for all $\left(h_{i},h_{j})\in \bar{E}(\bar{T}\right)$. Then, the problem of finding a phylogeny of H that satisfies the parsimony criterion consists of solving the following optimization problem:

#### Problem 2

The Most Parsimonious Phylogeny Estimation Problem from SNP haplotypes (MPPEP-SNP).Given a set H of *n* haplotypes having *m* alleles each, find the minimum cardinality haplotype set $H^{\prime }$ to be added to H so that the phylogeny ${\bar{T}}^{⋆}$ has minimum length.

If the haplotype set $H^{\prime }$ is empty, i.e., if G=(H,E) is connected, then MPPEP-SNP can be solved in polynomial time as the minimum spanning tree is a (optimal) solution to the MPPEP-SNP. Similarly, if the input haplotype set H satisfies the *perfect phylogeny condition* i.e., the requirement that each allele changes only once throughout the optimal phylogeny (see [19]), then the MPPEP-SNP can be still solved in polynomial time [26-28]. Unfortunately, it is possible to prove that in the general case the MPPEP-SNP is NP-hard (see [1,22]). In fact, the binary nature of the SNP haplotypes allows us to interpret a generic haplotype $h_{i}\in H$ as a vertex of a *m*-dimensional unit hypercube, its *s*-th allele as the *s*-th coordinate of the vertex *h*_*i*_, and the set $H^{\prime }$ as the set of Steiner vertices of the unit hypercube. Hence the MPPEP-SNP can be seen as particular case of the Steiner tree problem in a graph, a notorious NP-hard combinatorial optimization problem [29].

Finding the optimal solutions to the MPPEP-SNP is fundamental to validating the parsimony criterion, i.e., to verify whether, for a given instance of MPPEP-SNP, the results predicted by the criterion match the experimental ones. Unfortunately, the NP-hardness of the MPPEP-SNP limits the size of the instances analyzable to the optimum, which in turn affects the ability to validate the parsimony criterion, hence the practical utility of the predictions themselves. In order to address this concern, in the following section we shall develop an integer programming model able to provide optimal solutions to real instances of the MPPEP-SNP.

### A mixed integer programming model for the MPPEP-SNP

Let $V=\{1,2,\ldots ,n,n+1,n+2,\ldots ,n+|H^{\prime }|\}$ the set of potential vertices of a phylogeny $\bar{T}$ of H and assume the convention to denote the *n* haplotypes in H as the first *n* vertices in *V*. The first task in designing an integer programming model for the MPPEP-SNP that uses a polynomial-size number of variables consists of characterizing *V*, i.e., determining an upper and a lower bound on the cardinality of the set $H^{\prime }$. In fact, observe that $H^{\prime }$ contains potentially an exponential number of haplotypes, namely all vertices of the unit hypercube that belong to the set ${\left\{0,1\right\}}^{m}\setminus H$. However, we can easily bound the cardinality of $H^{\prime }$ by means of the following approach. Consider the complete graph Ĝ=(H,Ê), where $Ê=\{(h_{i},h_{j})\;:h_{i},h_{j}\in H\}$, and construct a minimum spanning tree $T_{Ĝ}$ of Ĝ. Denote $E(T_{Ĝ})$ as the set of edges (*h*_*i*_,*h*_*j*_) of $T_{Ĝ}$. Then, an upper bound UB on the overall number of Steiner vertices of the optimal phylogeny ${\bar{T}}^{⋆}$ can be obtained by computing the sum

$$\begin{array}{lcrr} \mathrm{UB}=\underset{\left(h_{i},h_{j})\in E(T_{Ĝ}\right)}{\sum }(d_{h_{i}h_{j}}-1). & & & \end{array}$$

Similarly, denote $L(T_{Ĝ})=\underset{\left(h_{i},h_{j})\in E(T_{Ĝ}\right)}{\sum }d_{h_{i}h_{j}}$, a lower bound LB on the overall number of Steiner vertices of ${\bar{T}}^{⋆}$ can be obtained as [30,31]:

$$\begin{array}{lcrr} \mathrm{LB}=\left\lceil \frac{L(T_{Ĝ})}{2}-n+1\right\rceil . & & & \end{array}$$

Denote *u*_*i*_, *i* ∈ *V*, as a decision variable equal to 1 if the *i*-th vertex of *V* is considered in the optimal solution to the MPEPP-SNP and 0 otherwise; $x_{i}^{s}$ as a decision variable equal to 1 if in the optimal solution to the MPPEP-SNP the *s*-th coordinate of the vertex *u*_*i*_, *i* ∈ *V*, is 1 and 0 otherwise; $z_{\text{ij}}^{s}$ as a decision variable equal to 1 if in the optimal solution to the MPPEP-SNP the pair of distinct vertices *i*,*j* ∈ *V* has a change at *s*-th coordinate, and 0 otherwise; and *y*_*ij*_ as a decision variable equal to 1 if the pair of distinct vertices *i*,*j*∈*V*is adjacent in the optimal solution to the MPPEP-SNP and 0 otherwise. Finally, let $V_{H}=\{1,2,\ldots ,n\}$, $V_{H^{\prime }}=\{n+1,n+2,\ldots ,n+\mathrm{UB}\}$, and *Q* = {1,2,…,*n* + *LB*}. Then, a valid formulation for the MPPEP-SNP is the following:

#### Formulation 1

Basic Model

(1a) $$\begin{array}{llll} \min & \;\underset{i,j\in V:i\neq j}{\sum }\underset{s\in S}{\sum }z_{\text{ij}}^{s}\; & \; & \; \end{array}$$

(1b) $$\begin{array}{lll} \mathrm{s.t.} & \;\;\;\;x_{i}^{s}=h_{i}(s)\; & \;\forall \;s\in S,i\in H\; \end{array}$$

(1c) $$\begin{array}{lll} & \;\;\;\;\;x_{i}^{s}\leq u_{i}\; & \;\forall \;s\in S,i\in V\; \end{array}$$

(1d) $$\begin{array}{lll} & \;z_{\text{ij}}^{s}\geq +x_{i}^{s}-x_{j}^{s}+y_{\text{ij}}-1\; & \;\forall \;s\in S,i,j\in V:i\neq j\; \end{array}$$

(1e) $$\begin{array}{lll} & \;z_{\text{ij}}^{s}\geq -x_{i}^{s}+x_{j}^{s}+y_{\text{ij}}-1\; & \;\forall \;s\in S,i,j\in V:i\neq j\; \end{array}$$

(1f) $$\begin{array}{lll} & \;\underset{s\in S}{\sum }z_{\text{ij}}^{s}=y_{\text{ij}}\; & \;\forall \;i,j\in V:i\neq j\; \end{array}$$

(1g) $$\begin{array}{lll} & \;y_{\text{ij}}\leq u_{i}\; & \;\forall \;i,j\in V:i\neq j\; \end{array}$$

(1h) $$\begin{array}{lll} & \;y_{\text{ij}}\leq u_{j}\; & \;\forall \;i,j\in V:i\neq j\; \end{array}$$

(1i) $$\begin{array}{lll} & \;\underset{j\in V:i\neq j}{\sum }y_{\text{ij}}\geq u_{i}\; & \;\forall \;i\in V\; \end{array}$$

(1j) $$\begin{array}{lll} & \;\underset{i,j\in C:i\neq j}{\sum }y_{\text{ij}}\leq \underset{i\in C}{\sum }u_{i}-1\; & \;\forall \;C\subset V:C\cap V_{H}\neq \emptyset \; \end{array}$$

(1k) $$\begin{array}{llll} & \;\underset{i,j\in V:i\neq j}{\sum }y_{\text{ij}}=\underset{i\in V}{\sum }u_{i}-1\; & \; & \; \end{array}$$

(1l) $$\begin{array}{llll} & \;\underset{i\in Q}{\sum }u_{i}=n+\mathrm{LB}\; & \; & \; \end{array}$$

(1m) $$\begin{array}{llll} & \;u_{i},\;x_{i}^{s},\;z_{\text{ij}}^{s},y_{\text{ij}}\in \{0,1\}.\; & \; & \; \end{array}$$

The objective function (1a) aims at minimizing the length of the optimal phylogeny. Constraints (1b) impose that the coordinates of the first *n* vertices in *V* are exactly the ones of the input haplotype set H. Constraints (1c) impose that the *s*-th coordinate of vertex *u*_*i*_, *i* ∈ *V*, can assume value 1 only if vertex *u*_*i*_ is considered in the optimal solution to the problem. Constraints (1d)-(1e) force variables $z_{\text{ij}}^{s}$ to be one if in the optimal solution to the problem there exist a pair of adjacent vertices *ij* ∈ *V* having a different value at the *s*-th coordinate. Constraints (1f) impose that in an optimal solution to the problem two distinct vertices *ij* ∈ *V* can be adjacent only if $d_{h_{i}h_{j}}=1$. Constraints (1g)-(1h) impose that in the optimal solution to the problem variable *y*_*ij*_ may assume value 1 only if both vertices *i* and *j* are considered. Vice versa, constraints (1i) impose that if in the optimal solution to the problem a vertex *u*_*i*_, *i* ∈ *V*, is considered then at least one variable *y*_*ij*_ must assume value 1. Constraints (1j) and (1k) impose the Generalized Subtour Elimination Constraints (GSEC) [23]. Finally, constraints (1l) impose that the first *n* + *LB*vertices in *V* have to be considered in the optimal solution to the problem.

Note that Formulation 1 can be easily extended to the case in which the haplotypes are represented by multi-character data, i.e., sequences over an alphabet *Σ* = {0,1,2,…,*γ*}. In fact, in such a case it is sufficient to replace each character *c* in the haplotype by a binary *γ* -vector *ν* such that the *s*-th coordinate of *ν* is equal to 1 if the character *c* is equal to *s*, *s* ∈ Σ, and 0 otherwise. For example, if the generic haplotype were, for example, the string 〈*AACGT*〉, then it could be represented as 〈1000 1000 0100 0010 0001〉.

### Reducing model size

Formulation 1 is characterized by a polynomial number of variables and an exponential number of constraints. Its implementation can be performed by means of standard branch-and-cut approaches that use GSEC separation oracles such as those described in [32].

It is worth noting that variables $x_{i}^{s}$ and $z_{\text{ij}}^{s}$ can be relaxed in Formulation 1c)-(1e) and the convexity constraint (1f) suffice to guarantee their integrality in any optimal solution to the problem. Moreover, Formulation 1 can be reduced in size by removing those variables that are redundant or whose value is known in the optimal solution to the problem. For example, variables *y*_*ij*_ can be removed from Formulation 1 as it is easy to realize that they are redundant. Similarly, all variables $z_{\text{ij}}^{s}$ such that $i,j\in V_{H}$ and *d*_*ij*_ > 1 do not need to be defined as their value will be always zero for any s∈S and in any feasible solution to the problem. Variables *u*_*i*_, *i* ∈ *Q*, do not need to be declared as their value will be always 1 any feasible solution to the problem. Finally, variables $x_{i}^{s}$, $i\in V_{H}$, can be removed as their value is univocally assigned by the input haplotype set H. The reduction process can be further combined with the preprocessing strategies described in [1] to obtain even smaller formulations. Such strategies allow one to remove alleles from the input haplotype set H without altering the optimal solution to the problem. For example, suppose that the haplotype set H is such that there exists an allele ŝ∈S such that $h_{i}(ŝ)=1$, for all $h_{i}\in H$; then it is easy to realize that ŝ can be removed from S as in any feasible solution to the problem the ŝ-th coordinate of any vertex in the phylogeny would be characterized by having *xiŝ* = 1. A similar situation occurs when there exists an allele ŝ∈S such that $h_{i}(ŝ)=0$, for all $h_{i}\in H$. Analogously, suppose that the input haplotype set H is characterized by *equal alleles*, i.e., by the existence of two alleles, say ${ŝ}_{1}$ and ${ŝ}_{2}$, such that $h_{i}({ŝ}_{1})=h_{i}({ŝ}_{2})$, for all i∈S. Then it is easy to realize that if one removes one of the two alleles from S, say ${ŝ}_{2}$, and multiplies the ${ŝ}_{1}$-th coordinate by 2 does not alter neither the structure nor the value of the optimal solution to the problem. Describing all the preprocessing techniques for shrinking the input haplotype set H is beyond the scope of the present article. The interested reader will find a systematic discussion of this topic in [1].

By applying the previously cited reduction strategies to Formulation 1 and denoting Ŝ as the set of alleles resulting from the application of the preprocessing strategies described in [1], *w*^*s*^ as the number of alleles in S equal to the *s*-th, s∈Ŝ, *Z* as the set for which variables $z_{\text{ij}}^{s}$ are defined, *R* = {*n* + *LB* + 1,*n* + *LB* + 2,…,*n* + *UB*}, and $C_{H}=\{i\in C:i\in V_{H}\}$, for any *C* ⊂ *V*, the following reduced formulation for the MPPEP-SNP can be obtained:

#### Formulation 2

Reduced Model

(2a) $$\begin{array}{cc} \min & \underset{\begin{array}{c} i,j\in V:\\ i,j\in Z \end{array}}{\sum }\;\underset{s\in Ŝ}{\sum }w^{s}z_{\text{ij}}^{s}\; \end{array}$$

(2b) $$\begin{array}{ccc} \mathrm{s.t.} & \;x_{i}^{s}\leq u_{i}\; & \;\forall \;s\in Ŝ,i\in R\; \end{array}$$

(2c) $$\begin{array}{cc} \underset{\begin{array}{c} s^{\prime }\in Ŝ:\\ s^{\prime }\neq s \end{array}}{\sum }z_{\text{ij}}^{s^{\prime }}+h_{i}(s)-x_{j}^{s}\leq 1\; & \;\forall \;s\in Ŝ,\;i\in V_{H},j\in V_{H^{\prime }}\; \end{array}$$

(2d) $$\begin{array}{cc} \underset{\begin{array}{c} s^{\prime }\in Ŝ:\\ s^{\prime }\neq s \end{array}}{\sum }z_{\text{ij}}^{s^{\prime }}-h_{i}(s)+x_{j}^{s}\leq 1\; & \;\forall \;s\in Ŝ,\;i\in V_{H},j\in V_{H^{\prime }}\; \end{array}$$

(2e) $$\begin{array}{cc} \underset{\begin{array}{c} s^{\prime }\in Ŝ:\\ s^{\prime }\neq s \end{array}}{\sum }z_{\text{ij}}^{s^{\prime }}+x_{i}^{s}-x_{j}^{s}\leq 1\; & \;\forall \;s\in Ŝ,\;i,j\in V_{H^{\prime }}:i,j\in Z\; \end{array}$$

(2f) $$\begin{array}{cc} \underset{\begin{array}{c} s^{\prime }\in Ŝ:\\ s^{\prime }\neq s \end{array}}{\sum }z_{\text{ij}}^{s^{\prime }}-x_{i}^{s}+x_{j}^{s}\leq 1\; & \;\forall \;s\in Ŝ,\;i,j\in V_{H^{\prime }}:i,j\in Z\; \end{array}$$

(2g) $$\begin{array}{cc} \underset{s\in Ŝ}{\sum }z_{\text{ij}}^{s}\leq 1\; & \;\forall \;i,j\in V\setminus R:i,j\in Z\; \end{array}$$

(2h) $$\begin{array}{c} \underset{s\in Ŝ}{\sum }z_{\text{ij}}^{s}\leq u_{i}\;\forall \;i\in R,j\in V:i,j\in Z\; \end{array}$$

(2i) $$\begin{array}{c} \underset{s\in Ŝ}{\sum }z_{\text{ij}}^{s}\leq u_{j}\;\forall \;j\in R,i\in V:i,j\in Z\; \end{array}$$

(2j) $$\begin{array}{c} \underset{\begin{array}{c} j\in V:\\ j\in Z \end{array}}{\sum }\underset{s\in Ŝ}{\sum }z_{\text{ij}}^{s}\geq 1\;\forall \;i\in Q\; \end{array}$$

(2k) $$\begin{array}{c} \underset{\begin{array}{c} j\in V:\\ j\in Z \end{array}}{\sum }\underset{s\in Ŝ}{\sum }z_{\text{ij}}^{s}\geq u_{i}\;\forall \;i\in R\; \end{array}$$

(2l) $$\begin{array}{cc} \underset{\begin{array}{c} i,j\in C:\\ i,j\in Z \end{array}}{\sum }\underset{s\in Ŝ}{\sum }z_{\text{ij}}^{s}\leq \underset{i\in C:i\in R}{\sum }u_{i}+|C_{H}|-1\\ & \forall \;C\subset V:C\cap V_{H}\neq \emptyset \; \end{array}$$

(2m) $$\begin{array}{l} \underset{\begin{array}{c} i,j\in V:\\ i,j\in Z \end{array}}{\sum }\underset{s\in Ŝ}{\sum }z_{\text{ij}}^{s}=n+\mathrm{LB}+\underset{i\in R}{\sum }u_{i}-1\; \end{array}$$

(2n) $$\begin{array}{l} u_{i},\;x_{i}^{s},\;z_{\text{ij}}^{s},y_{\text{ij}}\in \{0,1\}.\; \end{array}$$

Note that in Formulation 2 variables $x_{i}^{s}$ and $z_{\text{ij}}^{s}$ cannot be relaxed anymore.

### Strengthening valid inequalities

By exploiting the integrality of variables *u*_*i*_, $x_{i}^{s}$, and $z_{\text{ij}}^{s}$, a number of valid inequalities can be developed to strengthen Formulation 2.

#### Proposition 1

Constraints

(3) $$\begin{array}{lcrr} u_{i+1}\leq u_{i} & \; & \forall \;i\in V\setminus (Q\cup \{n+\mathrm{UB}\}) & \end{array}$$

are valid for Formulation 2.

#### Proof

In a feasible solution to the problem variable *u*_*i*_, *i* ∈ *V*∖(*Q* ∪ {*n* + *UB*}), can assume only value 0 or 1. If *u*_*i*_ = 0, constraint (3) reduces to *u*_*i* + 1_ ≤ 0 which is trivially valid for Formulation 2. If *u*_*i*_ = 1, constraint (3) reduces to *u*_*i* + 1_ ≤ 1 which is again valid. □

Constraints (3) impose an ordering on the variables *u*_*i*_, *i* ∈ *R*, so that vertex *u*_*i* + 1_ can be considered in the optimal solution to the problem only if vertex *u*_*i*_ has been already considered.

#### Proposition 2

Constraints

(4) $$\begin{array}{lcrr} \underset{\begin{array}{c} j\in V:\\ j\in Z \end{array}}{\sum }\;\underset{s\in Ŝ}{\sum }z_{\text{ij}}^{s}\geq 2u_{i} & \; & \forall \;i\in R & \end{array}$$

are valid for Formulation 2.

#### Proof

In a feasible solution to the problem a vertex *u*_*i*_, $i\in V_{H^{\prime }}$, cannot be a terminal vertex. In fact, if such a condition held, a cheaper solution could be obtained by dropping *u*_*i*_ from ${\bar{T}}^{⋆}$, contradicting the optimality of ${\bar{T}}^{⋆}$ itself. Hence, the degree of any vertex in $V_{H^{\prime }}$ must be at least 2. Now, in a feasible solution to the problem variables *u*_*i*_ ∈ {0,1}. If *u*_*i*_ = 0, constraint (4) reduces to

$$\underset{\begin{array}{c} j\in V:\\ j\in Z \end{array}}{\sum }\;\underset{s\in Ŝ}{\sum }z_{\text{ij}}^{s}\geq 0$$

which is trivially valid. Vice versa, if *u*_*i*_ = 1, constraint (4) reduces to

$$\underset{\begin{array}{c} j\in V:\\ j\in Z \end{array}}{\sum }\;\underset{s\in Ŝ}{\sum }z_{\text{ij}}^{s}\geq 2$$

which is again valid for the above arguments. □

#### Proposition 3

Constraints

(5) $$\begin{array}{lll} + & x_{i}^{s_{2}}-x_{j}^{s_{2}}\leq 2(1-z_{\text{ij}}^{s_{1}})-\underset{\begin{array}{c} \;s\in Ŝ:s\neq s_{1} \end{array}}{\sum }\;z_{\text{ij}}^{s}\; & \;\\ & \forall \;s_{1},s_{2}\in Ŝ:s_{1}\neq s_{2},\;i,j\in V_{H^{\prime }}:i,j\in Z\; \end{array}$$

(6) $$\begin{array}{lll} - & x_{i}^{s_{2}}+x_{j}^{s_{2}}\leq 2(1-z_{\text{ij}}^{s_{1}})-\underset{\begin{array}{c} \;s\in Ŝ:s\neq s_{1} \end{array}}{\sum }\;z_{\text{ij}}^{s}\; & \;\\ & \forall \;s_{1},s_{2}\in Ŝ:s_{1}\neq s_{2},\;i,j\in V_{H^{\prime }}:i,j\in Z\; \end{array}$$

are valid for Formulation 2.

#### Proof

As observed in the previous proposition, in a feasible solution to the problem $\underset{s\in Ŝ}{\sum }z_{\text{ij}}^{s}$, $i,j\in V_{H^{\prime }}$, *i*,*j*∈*Z*, can assume only value 0 or 1. If $\underset{s\in Ŝ}{\sum }z_{\text{ij}}^{s}=0$, then constraint (5) (respectively constraint (6)) reduces to $+x_{i}^{s_{2}}-x_{j}^{s_{2}}\leq 2$ (respectively $-x_{i}^{s_{2}}+x_{j}^{s_{2}}\leq 2$), which is trivially valid due to the integrality of variables $x_{i}^{s}$. If $\underset{s\in Ŝ}{\sum }z_{\text{ij}}^{s}=1$, then either $\underset{\begin{array}{c} s\in Ŝ:\\ s\neq s_{1} \end{array}}{\sum }z_{\text{ij}}^{s}=1$ or $z_{\text{ij}}^{s_{1}}=1$. If $\underset{\begin{array}{c} s\in Ŝ:\\ s\neq s_{1} \end{array}}{\sum }z_{\text{ij}}^{s}=1$ then constraint (5), (respectively constraint (6)) reduces to $+x_{i}^{s_{2}}-x_{j}^{s_{2}}\leq 1$ (respectively $-x_{i}^{s_{2}}+x_{j}^{s_{2}}\leq 1$), which is trivially valid. If $z_{\text{ij}}^{s_{1}}=1$ then constraint (5) (respectively constraint (6)) reduces to $+x_{i}^{s_{2}}-x_{j}^{s_{2}}\leq 0$ (respectively $-x_{i}^{s_{2}}+x_{j}^{s_{2}}\leq 0$), which is again valid. □

Similar arguments can be used to prove the following proposition:

#### Proposition 4

Constraints

(7) $$\begin{array}{lll} + & h_{i}(s_{2})-x_{j}^{s_{2}}\leq 2(1-z_{\text{ij}}^{s_{1}})-\underset{\begin{array}{c} s\in Ŝ:\\ s\neq s_{1} \end{array}}{\sum }z_{\text{ij}}^{s}\; & \;\\ & \forall \;s_{1},s_{2}\in Ŝ:s_{1}\neq s_{2},\;i\in V_{H},\;j\in V_{H^{\prime }}\; \end{array}$$

(8) $$\begin{array}{lll} - & h_{i}(s_{2})+x_{j}^{s_{2}}\leq 2(1-z_{\text{ij}}^{s_{1}})-\underset{\begin{array}{c} s\in Ŝ:\\ s\neq s_{1} \end{array}}{\sum }z_{\text{ij}}^{s}\; & \;\\ & \forall \;s_{1},s_{2}\in Ŝ:s_{1}\neq s_{2},\;i\in V_{H},\;j\in V_{H^{\prime }}\; \end{array}$$

are valid for Formulation 2.

**Figure 1:**
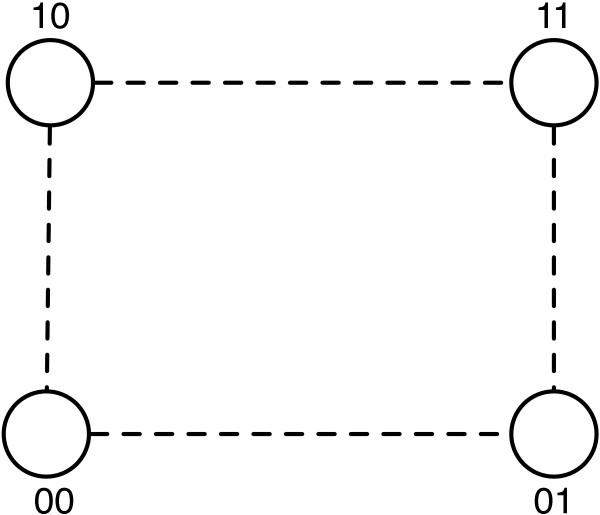
**An example of two symmetric paths linking haplotypes*〈*00*〉*and*〈*11*〉*.**

Given an input haplotype set H and a pair of non-adjacent haplotypes *h*_*i*_ and *h*_*j*_, there exit multiple equivalent paths that may connect *h*_*i*_ and *h*_*j*_ in the unary hypercube. This characteristic constitutes the principal class of symmetries for the MPPEP-SNP and may lead to poor relaxations for the problem. For example, suppose that the input haplotype set H is constituted by haplotypes *h*_1_ = 〈00〉 and *h*_2_ = 〈11〉. Then a possible solution to the instance may consist either of a star centered in haplotype *h*_3_ = 〈10〉 or a star centered in haplotype *h*_3_ = 〈01〉(see Figure 1). Note that both solutions are feasible and optimal for the specific instance. A possible strategy to break this class of symmetries consists of imposing the following constraints:

#### Proposition 5

Constraints

(9) $$\begin{array}{lll} & \overset{s}{\underset{p=1}{\sum }}2^{s-p}x_{i}^{p}\leq \overset{s}{\underset{p=1}{\sum }}2^{s-p}x_{i+1}^{p}\; & \;\\ & \;\forall \;s\in Ŝ,i\in V_{H^{\prime }}\setminus R\; \end{array}$$

(10) $$\begin{array}{lll} & \overset{s}{\underset{p=1}{\sum }}2^{s-p}x_{i}^{p}\leq \overset{s}{\underset{p=1}{\sum }}2^{s-p}x_{i+1}^{p}+\overset{s}{\underset{p=1}{\sum }}2^{s-p}(1-u_{i+1})\; & \;\\ & \;\forall \;s\in Ŝ,i\in R\setminus \{n+\mathrm{UB}\}\; \end{array}$$

are valid for Formulation 2.

#### Proof

The statement trivially follows from the integrality of variables $x_{i}^{s}$ and from constraints (2b). □

Constraints (9)-(10) impose an ordering on the coordinates of the vertices in $V_{H^{\prime }}$ by means of the smallest big-M possible, i.e., a power of 2. Note that the distinction between constraints (9) and (10) is necessary, as in principle vertices in *R* may not be needed in the optimal solution to the problem.

#### Proposition 6

Constraints

(11) $$\begin{array}{lcrr} \underset{\begin{array}{c} j\in V:\\ j\in Z \end{array}}{\sum }\;\underset{s\in Ŝ}{\sum }z_{\text{ij}}^{s}\geq \underset{\begin{array}{c} j\in V:\\ j\in Z \end{array}}{\sum }\;\underset{s\in Ŝ}{\sum }z_{(i+1)j}^{s} & & \forall i\in V_{H^{\prime }}\setminus \{n+\mathrm{UB}\} & \end{array}$$

are valid for Formulation 2.

#### Proof

In a feasible solution to the problem, the sum $\underset{s\in Ŝ}{\sum }z_{\text{ij}}^{s}$, $i,j\in V_{H^{\prime }}$, *i*,*j*∈*Z*, can assume only value 0 or 1. If $\underset{\begin{array}{c} j\in V:\\ j\in Z \end{array}}{\sum }\;\underset{s\in Ŝ}{\sum }z_{(i+1)j}^{s}=0$, constraint (11) reduces to $\underset{\begin{array}{c} j\in V:\\ j\in Z \end{array}}{\sum }\;\underset{s\in Ŝ}{\sum }z_{\text{ij}}^{s}\geq 0$ which is trivially valid. Vice versa, If $\underset{\begin{array}{c} j\in V:\\ j\in Z \end{array}}{\sum }\;\underset{s\in Ŝ}{\sum }z_{(i+1)j}^{s}=1$, constraint (11) reduces to $\underset{\begin{array}{c} j\in V:\\ j\in Z \end{array}}{\sum }\;\underset{s\in Ŝ}{\sum }z_{\text{ij}}^{s}\geq 1$ which is again valid due to Propositions 1 and 2. □

**Figure 2:**
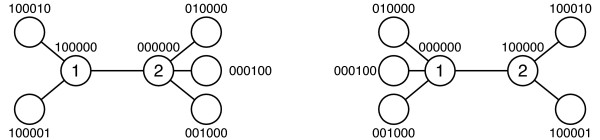
An example of two symmetric solution to the MPPEP-SNP.

Proposition 6 forces vertices in $V_{H^{\prime }}$ to be sorted according to a decreasing degree order. In this way, it is possible to avoid the occurrence of symmetric solutions to the problem differing just for the degree of the Steiner vertices (see e.g., Figure 2).

Let $Q_{3}=\{i,j\in V_{H}:d_{\text{ij}}\geq 3\}$ and *k* ∈ *V*, *k* ∉ *Q*_3_. Then the following proposition holds:

#### Proposition 7

Constraints

(12) $$\begin{array}{lcrr} \underset{s\in S}{\sum }z_{\text{ik}}^{s}+\underset{s\in S}{\sum }z_{\text{kj}}^{s}\leq 1 & \; & \forall \;i,j\in Q_{3} & \end{array}$$

are valid for Formulation 2.

#### Proof

In a feasible solution to the problem the path between two distinct haplotypes $h_{i},h_{j}\in H$ cannot be shorter than the distance $d_{h_{i}h_{j}}$. Hence, if the distance between *h*_*i*_and *h*_*j*_is greater or equal to three, vertices *i* and *j* cannot be adjacent to a same vertex *k*, i.e., only one of the two sums $\underset{s\in S}{\sum }z_{\text{ik}}^{s}$ or $\underset{s\in S}{\sum }z_{\text{jk}}^{s}$ can be equal to 1. □

Note that if *k* ∈ *R* then (12) can be strengthened by replacing the right-hand-side by *u*_*k*_. Moreover, Proposition 7 can be generalized as follows. Consider the sets $Q_{d}=\{i,j\in V_{H}:d_{\text{ij}}\geq d\}$, *d* ∈ {3,4,…,*m*}, *C* ⊂ *V* such that 2 ≤ |*C*| ≤ *d* − 1 and *C* ∩ *Q*_*d*_ = *∅*, and a path *p* that involves only vertices in *C*. Denote *p*_*k*_ the *k*-th vertex in *p*. Then the following proposition holds:

#### Proposition 8

The family of constraints

(13) $$\begin{array}{lcrr} \underset{s\in S}{\sum }z_{{\mathrm{ip}}_{1}}^{s}\;+\;\overset{|C|-1}{\underset{k=1}{\sum }}\underset{s\in S}{\sum }z_{p_{k}p_{k+1}}^{s}\;+\;\underset{s\in S}{\sum }z_{p_{|C|j}}^{s}\;\leq \;|C| & \; & \;\forall \;i,j\in K_{d}, & \end{array}$$

called forbidden path constraints, are valid for Formulation 2.

#### Proof

Similarly to Proposition 7, in a feasible solution to the problem the path *p* between two distinct haplotypes $h_{i},h_{j}\in H$ cannot be shorter than the distance $d_{h_{i}h_{j}}$. Hence, if the distance between *h*_*i*_ and *h*_*j*_ is greater or equal to *d*, at most |*C*| vertices can belong to *p*. □

## Experiments

In this section we analyze the performance of our model to solve the MPPEP-SNP. Our experiments were motivated by a twofold reason, namely: to evaluate, with respect to Formulation 1, the benefits obtained by the removal of the redundant variables and by the inclusion of the valid inequalities previously described; and to allow the analysis of larger datasets with respect to the ones analyzable by means of [1]’s algorithm, currently the best known exact approach to solution of the MPPEP-SNP.

Similar to [1], we emphasize that the experiments aim simply to evaluate the runtime performance of our model for solving MPPEP-SNP. We neither attempt to study the efficiency of MPPEP-SNP for phylogeny estimation nor compare the accuracy of our algorithm to phylogeny estimation solvers that do not use the parsimony criterion. The reader interested in a systematic discussion about such issues is referred to [19,33].

### Implementation

We implemented Formulations 1 and 2 by means of Mosel 64 bit 3.2.0 of Xpress-MP, Optimizer version 22, running on a Pentium 4, 3.2 GHz, equipped with 2 GByte RAM and operating system Gentoo release 7 (kernel linux 2.6.17). In both formulations, we computed the overall solution time to solve a generic instance of the problem as the sum of the preprocessing time due to the implementation of [22]’s reduction rules plus the solution time taken by the Optimizer to find the optimal solution to the instance. In preliminary experiments, we observed that Formulation 2 has two main advantages with respect to Formulation 1, namely: it requires much less memory to load the model (at least 27% RAM less in the analyzed instances) and it is characterized by faster linear relaxations at each node of the search tree. As result, Formulation 2 allows potentially the analysis of larger instances than Formulation 1 and may be characterized by faster solution times. Hence, we preferred to use Formulation 2 in our experiments.

We considered two different implementations of Formulation 2, namely: the GESC-based Reduced Model (GSEC-RM) and the Flow-based Reduced Model (Flow- RM). The GESC-RM consists of Formulation 2 strengthened by the valid inequalities previously described. The Flow-RM consists of Formulation 2 strengthened by the valid inequalities and such that the GSEC are replaced by a multi-commodity flows. Specifically, by denoting $f_{\mathrm{ij}}^{q}$ as a decision variable equal to one if there exists a flow from vertex 1 to vertex $q\in V_{H}$ passing through edge $(\mathrm{i,j}\in \overset{¯}{E}$ and 0 otherwise, the Flow-RM can be obtained by replacing constraints 2l) with:

(14) $$\begin{array}{lcrr} f_{\text{ji}}^{q}\;+\;f_{\text{ij}}^{q}\;\leq \;\underset{s\in S}{\sum }z_{\text{ij}}^{s} & \;\; & \forall \;q\;\in \;V_{H}:q\;\neq \;1,\;i,j\;\in \;V:i,j\in Z & \end{array}$$

(15) $$\begin{array}{lcrr} \underset{i\in V:i\neq 1}{\sum }f_{1i}^{q}=1 & \;\; & \forall \;q\in V_{H}:q\neq 1 & \end{array}$$

(16) $$\begin{array}{lcrr} \underset{j\in V:i\neq j}{\sum }f_{\text{ij}}^{q}\;-\;\underset{j\in V:i\neq j}{\sum }f_{\text{ji}}^{q}\;=\;0 & \;\; & \forall \;q\;\in \;V_{H}:q\;\neq \;1,\;i\in V:i\notin \{1,q\} & \end{array}$$

(17) $$\begin{array}{lcrr} \underset{i\in V:i\neq q}{\sum }f_{\text{iq}}^{q}-\underset{i\in V:i\neq q}{\sum }f_{\text{qi}}^{q}=1 & \;\; & \forall \;q\in V_{H}:q\neq 1 & \end{array}$$

(18) $$\begin{array}{lcrr} f_{\text{ij}}^{q}\;\geq \;0 & \;\; & \forall \;q\;\in \;V_{H}:q\;\neq \;1,\;i,j\;\in \;V:i,j\;\in \;\mathrm{Z.} & \end{array}$$

In preliminary experiments we observed that the Flow-RM outperforms the GESC-RM in terms of solution time. This fact is mainly due to the computational overhead introduced by the GSEC separation oracle which seems to be not compensated by the size of the analyzed instances. Hence, we did not consider the GESC-RM any further in our experiments.

During the runtime, we enabled the Xpress-MP automatic cuts and the Xpress-MP pre-solving strategy. Moreover, we also tested a number of generic primal heuristics for the Steiner tree problem to generate a first primal bound to the MPPEP-SNP (see, e.g., [34]). Unfortunately, in preliminary experiments we observed that the use of such heuristics interferes negatively with the Xpress Optimizer, by delaying the solution time of the analyzed instances. Hence, we disabled the used of the generic primal heuristics and enabled the use of the Xpress-MP primal heuristic instead. The source code of the algorithm can be downloaded at http://homepages.ulb.ac.be/~dacatanz/Site/Software_files/iMPPEP.zip.

### Separation oracle for the forbidden path constraints

When using the Flow-RM, the valid inequalities (3)-(12) are loaded together with the reduced model. On the contrary, the valid inequalities (13) are dynamically generated by means of a separation oracle working as follows. Before loading the reduced model, we precompute the sets *Q*_*d*_, for all *d* ∈ {3,4,…,*m*}. Let $\left(\bar{u},\bar{x},\bar{z}\right)$ be the current fractional solution at a given node of the search tree and, for all *d* ∈ {3,4,…,*m*}, consider a pair of vertices *i*,*j*∈*Q*_*d*_. Then, the forbidden path constraints (13) are violated if there exists a path *p* having internal vertices in *C* ⊂ *V*, 2 ≤ |*C*| ≤ *d* − 1, *C* ∩ *Q*_*d*_ = *∅*, and such that

(19) $$\begin{array}{lcrr} \underset{s\in S}{\sum }{\bar{z}}_{{\mathrm{ip}}_{1}}^{s}+\overset{|C|-1}{\underset{k=1}{\sum }}\underset{s\in S}{\sum }{\bar{z}}_{p_{k}p_{k+1}}^{s}+\underset{s\in S}{\sum }{\bar{z}}_{p_{|C|j}}^{s}>|C|. & & & \end{array}$$

Note that searching for the most violated constraint (19) is in general NP-hard as it involves solving a longest path problem on the weighted graph ${\bar{G}}_{\bar{z}}^{V\setminus Q_{d}}$, i.e., the graph $\bar{G}$ whose edges are weighted by variables $\bar{z}$ and whose vertex set is restricted to (*V*∖*Q*_*d*_) ∪ {*i*,*j*}. In order to have a fast separation oracle for the forbidden path constraints we do not solve exactly (19) but we use a heuristic approach instead. Specifically, we first sort edges of $\bar{E}$ in decreasing order according to their weights and we select two distinct vertices *v*_1_,*v*_2_ ∈ *V*∖*Q*_*d*_ such that edge (*v*_1_,*v*_2_) has the largest weight. Subsequently, we set *C* = {*v*_1_,*v*_2_}, mark *v*_1_ and *v*_2_ as visited, and build a simple path from vertex *i* to vertex *j* passing by *v*_1_ and *v*_2_. If *p* is such that (19) is satisfied then we add the constraint

(20) $$\begin{array}{lcrr} \underset{s\in S}{\sum }z_{{\mathrm{ip}}_{1}}^{s}+\overset{|C|-1}{\underset{k=1}{\sum }}\underset{s\in S}{\sum }z_{p_{k}p_{k+1}}^{s}+\underset{s\in S}{\sum }z_{p_{|C|j}}^{s}\leq |C| & & & \end{array}$$

to the formulation; otherwise, we select a different pair of vertices in *V*∖*Q*_*d*_ and iterate this procedure until either 10 distinct paths have been generated or all possible pairs of vertices in *V*∖*Q*_*d*_ have been selected. If all vertices have been selected but less than 10 distinct paths have been generated, then we select a larger subset of *V*∖*Q*_*d*_ (e.g., a triplet of vertices) and we iterate again the previous steps. It is easy to realize that this procedure may potentially generate all the possible paths violating (13). However, we stop the procedure after generating 10 paths or after considering subset *C* containing more than 5 vertices as we observed in preliminary experiments that this strategy provides the best trade-off between speed and tightness of the cut.

### Branching strategies

In preliminary experiments we observed that the standard branching strategy implemented in the Xpress-MP Optimizer is not appropriate for the problem as it is not able to exploit the dissimilarity of the weights *w*^*s*^ in the objective function. This inconveniently leads to formulations characterized by slow solution times. To improve this aspect we implemented a different strategy consisting of branching on the following constraints:

(21) $$\begin{array}{lcrr} \underset{\begin{array}{c} i,j\in V:\\ i,j\in Z \end{array}}{\sum }\;z_{\text{ij}}^{s}\leq \alpha & \; & \forall \;s\in S & \end{array}$$

or

(22) $$\begin{array}{lcrr} \underset{\begin{array}{c} i,j\in V:\\ i,j\in Z \end{array}}{\sum }\;z_{\text{ij}}^{s}>\alpha , & \; & \forall \;s\in S & \end{array}$$

where *α* ∈ {1,2,…,*q*} and $q=\min\{\underset{k\in V_{H}}{\sum }h_{k}(s),n/2\}$. Constraints (21)-(22) limit the number of changes along a phylogeny with respect to a given coordinate s∈S and tend to be more effective when the weights *w*^*s*^ are very dissimilar among them. This branching strategy can be implemented by introducing a decision variable

$$\begin{array}{cccc} \beta_{\alpha }^{s}=\left\{\begin{array}{c} 1\text{if the overall number of changes at coordinate}\\ \;s\in S\;\text{of}\;{\bar{T}}^{⋆}\;\text{is equal to}\alpha \\ 0\;\text{otherwise}, \end{array}\right) & & & \end{array}$$

for all s∈S and *α* ∈ {1,2,…,*q*}, and by adding the following constraints

$$\begin{array}{lcrr} \underset{\begin{array}{c} i,j\in V:\\ i,j\in Z \end{array}}{\sum }z_{\mathrm{ij}}^{s}=\overset{q}{\underset{\alpha =1}{\sum }}\beta_{\alpha }^{s} & \; & \forall \;s\in S & \\ \;\overset{q}{\underset{\alpha =1}{\sum }}\beta_{\alpha }^{s}=1 & \; & \forall \;s\in S. & \end{array}$$

We observed that even better runtime performance can be obtained by sorting the coordinates of the input haplotypes in decreasing way according to the weights *w*^*s*^ and by branching first on variables $\beta_{\alpha }^{s}$, then on variables *u*_*i*_, and subsequently on variables $x_{i}^{s}$ and finally on variables $z_{\mathrm{ij}}^{s}$.

### Performance analysis

In order to obtain a measure of the performance of the Flow-RM, we compared [1]’s polynomial-size formulation versus the Flow-RM on [1]’s real instances of the MPPEP-SNP, namely: Human chromosome Y constituted by 150 haplotypes having 49 SNPs each; bacterial DNA constituted by 17 haplotypes having 1510 SNPs each; Chimpanzee mitochondrial DNA constituted by 24 haplotypes having 1041 SNPs each; Chimpanzee chromosome Y constituted by 24 haplotypes having 1041 SNPs each; and a set of four Human mitochondrial DNA from HapMap [35] constituted by 40 haplotypes having 52 SNPs each, 395 haplotypes having 830 SNPs each, 13 haplotypes having 390 SNPs each, and 44 haplotypes having 405 SNPs each, respectively. Such instances consist only of non-recombining data (Y chromosome, mitochondrial, and bacterial DNA) and can be downloaded at http://homepages.ulb.ac.be/~dacatanz/Site/Software_files/iMPPEP.zip.

Table 1 shows the results obtained by such comparison. Specifically, the fourth and fifth columns refer to the gaps (expressed in percentage) of the respective formulations, i.e., to the difference between the optimal value to a specific instance and the value of linear relaxation at the root node of the search tree, divided by the optimal value. The table shows that, excluding the cases in which the solution to a specific instance was trivially a minimum spanning tree (see e.g., Human chromosome Y, Chimpanzee mtDNA, and Chimpanzee chromosome Y), the Flow-RM is always characterized by (sometimes dramatically) smaller gaps. This fact derives on the one hand from the tightness of the Flow-RM with respect to [1]’s polynomial-size formulation and on the other hand from the efficiency of the strengthening valid inequalities previously described. The poor relaxations of their formulation led [1] to propose an alternative and faster exact approach to solution of the MPPEP-SNP based on the brute-force enumeration of all possible Steiner vertices necessary to solve a specific instance of the problem. To speed up the computation, the brute-force enumeration algorithm makes use of a set of reduction rules based on Buneman graph enumeration to decrease the number of Steiner vertices to be considered. Interestingly, despite the differences in terms of implementation language between the two programs (namely, Mosel for the Flow-RM and C++ for [1]’s brute-force enumeration algorithm), the Flow-RM proved to be competitive with [1]’s enumeration algorithm, being able to solve almost all the considered instances within 1 second computing time. Only in two cases, namely Human mtDNA 40×52 and Human mtDNA 395×830, the Flow-RM needed more than 5 minutes to find the corresponding optimal solutions. The deterioration of the runtime performance in those instances is mainly due to the overhead necessary to load the formulation (that in both cases is considerably bigger than in the other instances) and to an intensive use of the separation oracle for the forbidden path constraints. Possibly, a more thorough implementation of the separation oracle and the use of more performing languages (e.g., C++) could help in speeding up computations in those instances at least. 

**Table 1:** **Comparison between the gap of [**[1]**]’s polynomial size integer programming model for the MPPEP-SNP versus the gap of the flow-based reduced model and its strengthening valid inequalities**

**Dataset**	**Haplotypes**	**SNPs**	**GAP (%)**	**GAP (%)**	**Optimum**	**MST**
			**[**[1]**]**	**Flow-RM**		**Solution**
Human chromosome Y	150	49	0.00	0.00	16	yes
Bacterial mtDNA	17	1510	26.04	**20.83**	96	no
Chimpanzee mtDNA	24	1041	20.63	20.63	63	yes
Chimpanzee chromosome Y	15	98	0.00	0.00	99	yes
Human mtDNA	40	52	24.66	**1.37**	73	no
Human mtDNA	395	830	22.64	**7.55**	53	no
Human mtDNA	13	390	12.50	**6.25**	48	no
Human mtDNA	44	405	6.98	**4.65**	43	no

Interestingly, sometimes in real applications the number of haplotypes can be much bigger than the number of SNPs. Hence, it is important to test the ability of an exact algorithm to tackle instances of the MPPEP-SNP containing e.g., hundreds haplotypes. [1] observed that their brute force enumeration algorithm is able to tackle instances of the problem containing up to 270 haplotypes having up to 9 SNPs each within 12 hours computing time. Unfortunately, the authors also observed that their algorithm is unable to solve larger instances of the MPPEP-SNP, no matter the maximum runtime considered. In this context, the Flow-RM makes the difference, being able to tackle instances of the MPPEP-SNP having up to 300 haplotypes and 10 SNPs within 3 hours computing time. To show this result, we considered a number of random instances of the problem containing 100, 150, 200, 250, and 300 haplotypes, respectively. Fixing the number of haplotypes *n*∈{100,150,200,250,300}, we created an instance of the problem by generating at random *n* strings of length 10 over the alphabet *Σ*={0,1}. During the generation process, we randomly selected the number of SNPs equal to 1 in a given haplotype, and subsequently we randomly chose the sites of the haplotype to be set to 1. We iterated the instance generation process 10 times for a fixed value of *n*, obtaining eventually an overall number of 50 random instances of the MPPEP-SNP downloadable at http://homepages.ulb.ac.be/~dacatanz/Site/Software_files/iMPPEP.zip.

The results obtained in our experiments are shown in Table 2. Specifically, the column “Time” refers to the solution time (expressed in seconds) necessary to solve exactly a specific instance of the MPPEP-SNP. Analogously, the column “Nodes” refers to the number of explored nodes in the search tree needed to solve exactly the instance. The table does not report on the performance of [1]’s enumeration algorithm, as their algorithm never found the optimal solution to the analyzed instances within the limit runtime of 3 hours. As a general trend, the table shows that the considered instances can be exactly solved within 1 hour computing time. The only exceptions are constituted by the 7th instance of the group 150×10, the 9th instance of the group 200×10, the 2th instance of the group 250×10, and 3th instance of the group 300×10which needed 8719.65, 4600.69, 7757.98, and 5371.05 seconds, respectively, to be solved. These instances are much more sparse than the others, are characterized by smaller reduction ratios, and tend to have more degenerate relaxations than the other instances. The presence of these factors might explain the loss of performance of the Flow-RM. 

**Table 2:** Performances of the Flow-RM on a set of random instances of the MPPEP-SNP

$\left|H\right|\;$	**Instance**	$\left|H\right|\;$**post**	**Time**	**GAP (%)**	**Nodes**	$\left|H\right|\;$	**Instance**	$\left|H\right|\;$**post**	**Time**	**GAP (%)**	**Nodes**
		**reduction**	**(sec.)**					**reduction**	**(sec.)**		
100	1	57	520.05	0	1807	150	1	82	284.51	0	424
	2	60	59.74	0	174		2	83	314.27	0.76	56
	3	63	377.75	1.45	110		3	81	799.01	0	67
	4	61	2491.62	3.81	3351		4	67	1809.26	2.66	6617
	5	60	2918.09	4.63	2062		5	79	1001.14	2.29	187
	6	57	349.54	1.59	264		6	74	1976.73	2.41	1071
	7	65	258.53	1.90	85		7	73	8719.65	3.92	4814
	8	58	293.97	0	1299		8	83	3497.73	2.17	421
	9	62	862.48	2.85	540		9	72	1154.77	2.51	410
	10	64	87.19	0	92		10	80	399.89	1.56	256
200	1	99	614.86	0	72	250	1	117	1155.41	0	197
	2	99	1353.16	1.28	149		2	109	7757.98	1.72	1596
	3	96	896.68	0.67	226		3	117	387.141	0.84	180
	4	104	652.44	0.47	150		4	126	1267.77	0.51	114
	5	96	382.83	0	56		5	116	188.188	0.84	162
	6	106	2535.09	0.60	71		6	116	2311.61	1.14	685
	7	100	233.50	0	21		7	116	1256.24	0	265
	8	99	1650.17	0.96	79		8	124	67.556	0	528
	9	87	4600.69	2.10	954		9	122	2000.77	0.53	107
	10	102	2554.84	1.23	1965		10	111	1200.89	0.87	272
300	1	133	297.19	0	15						
	2	123	2753.53	0.39	68						
	3	142	5371.05	0	941						
	4	133	420.72	0	43						
	5	126	388.99	0	433						
	6	134	397.01	0	61						
	7	138	1173.65	0	1788						
	8	126	666.21	0	186						
	8	127	449.30	0.77	42						
	10	145	201.87	0	876						

The results showed that the integrality gaps are usually very low, ranging from 0% to 4.63% and assuming in average a value about 1%, confirming once again the tightness of the Flow-RM and the efficiency of the strengthening valid inequalities.

Finally, we also tested the performance of the Flow-RM on a set of 5 HapMap Human mitochondrial DNA instances of the MPPEP-SNP that were not solvable by using [1]’s brute-force enumeration algorithm, namely: f1 constituted by 63 haplotypes having 16569 SNPs each, i2 constituted by 40 haplotypes having 977 SNPs each, k3 constituted by 100 haplotypes having 757 SNPs each, m4 constituted by 26 haplotypes having 48 SNPs each, and p5 constituted by 21 haplotypes having 16548 SNPs each. Such instances can be downloaded at the same address and consist only of non-recombining data (Y chromosome, mitochondrial, and bacterial DNA).

A part from m4, all the remaining instances gave rise to too large formulations (several hundreds Mbytes RAM) to be handled by the Xpress Optimizer. Hence, instead of analyzing entirely each instance we decomposed it into contiguous SNP blocks and analyzed the most difficult block separately. In more in detail, we define $H_{r}$ to be the haplotype matrix obtained by the application of [1]’s reduction rules, we sorted the columns of $H_{r}$ according to an increasing ordering of the weights *w*^*s*^, s∈Ŝ; subsequently, we considered the submatrices obtained by taking *k* contiguous SNPs (or *k*-block) in Ŝ,*k* ∈ {10,13,15}. We did not consider greater values for *k* as we observed that *k* = 15 was already a threshold after which the haplotype submatrix gave rise to too large formulations. For each *k*-block B in $H_{r}$ we considered the hamming distance $d_{h_{i}h_{j}}=\underset{s\in B}{\sum }|h_{i}(s)-h_{j}(s)|$ between each pair of distinct haplotypes in $H_{r}$, and chose the *k*-block maximizing the sum $\underset{h_{i},h_{j}\in H_{r},h_{i}<h_{j}}{\sum }d_{h_{i}h_{j}}$. Finally, we assumed three hours as maximum runtime per instance.

Table 3 shows the results obtained in our experiments. As for Table 2, the columns “Time” and “Nodes” refer to the solution time (expressed in seconds) and to the number of nodes in the search tree necessary to solve exactly a specific instance of the MPPEP-SNP, respectively. In such a case, the values in the columns “Gap” refers to the gap between the best primal bound found within the limit time and the root relaxation and “Nodes” refers to the number of nodes explored in the tree search within the limit time.

**Table 3:** Performances of the Flow-RM on a set of real instances of the MPPEP-SNP

**Dataset**	**Haplotypes**	**SNPs**	**Block Size**	**Time (sec.)**	**GAP (%)**	**Nodes**	**MST Solution**
			10	1	|	|	yes
f1	63	16569	13	56	3.11	1	no
			15	10286.1	26.92	773521	no
i2	40	977	10	781.85	20.00	37511	no
k3	100	757	10	150	7.65	353	no
			13	588.38	14.29	11265	no
m4	26	48	10	5	5.88	109	no
p5	21	16548	10	22283.4	50.79	6125448	no

Table 3 shows that, apart from the instances f1 and m4, the Flow-RM was unable to exactly solve, within the limit time, the considered instances for values of *k* ∈ {13,15}. Specifically, The Flow-RM exactly solved in less than a minute the instance f1 when considering values of *k* ∈ {10,13} ; in 20 minutes the instance i2 when considering *k* = 10 ; in less than 3 minutes the instance k3 when considering *k* = 10; and the instance m4 in 5 seconds. In contrast, the Flow-RM was unable to solve the instance p5, regardless of the value of *k* considered. In fact, already when considering *k* = 10, the Xpress Optimizer took more than 12 hours to exactly solve the instance p5 and explored over 10 million nodes in the search tree. A more detailed analysis of the instance showed that, despite the presence of the strengthening valid inequalities, p5 is characterized by highly fractional relaxations. This fact implies the existence of equivalent optimal solutions to the instance that, on the one hand, delay the finding of a primal bound and, on the other hand, force the Optimizer to explore many more nodes in the tree search. This situation in more pronounced in p5 but also occurs in the instances i2 and k3. To improve the tightness of the formulation we tried to include in the Flow-RM also classical facets and strengthening valid inequalities for the Steiner tree problem in a graph (see [23,36-38]). However, we did not observe any benefit from the inclusion. We suspect that the presence of highly fractional solutions to the problem could be caused both by poor lower bounds on the number of Steiner vertices considered in the Flow-RM and by the existence of a number of non trivial classes of symmetries still present in the problem. Investigating such issues warrants future research efforts.

In order to measure the performance of the model on multi-state character data we also considered [2] set of instances of the MPPEP-SNP. Specifically, we considered the following instances: a set of 41 sequences of O.rufipogon DNA (red rice) having 1043 sites each; 80 human mtDNA sequences having 245 sites each; 50 HIV-1 reverse transcriptase amino acid sequences having 176 sites each; a set of 500 sequences of mtDNA from the NCBI BLASTN best aligned taxa having 3000 sites each; a set of 500 sequences of mtDNA from the NCBI BLASTN best aligned taxa having 5000 sites each; and a set of 500 sequences of mtDNA from the NCBI BLASTN best aligned taxa having 10000 sites each. When running the same experiments described in [2] we registered a very poor performance for the Flow-RM, mainly due to the large dimension of the considered instances and the presence of symmetries despite the use of constraints (13)-(15). We observed that the combination of these two factors increased the runtime performance of the Flow-RM of 2-3 orders of magnitude with respect to [2] approach. However, we also observed that when performing [2]’s “window analysis” (i.e., when decomposing into blocks of 10 SNPs the input matrix) the Flow-RM performed better than [2]’s, being characterized by an average solution time of 8.27 seconds. This fact suggests that, when considering instances constituted by less than a dozen sites, an exact approach entirely based on integer programming may perform better than the implicit enumeration of the vertices of the generalized Buneman graph. Vice-versa, for larger instances the implicit enumeration of the vertices of the generalized Buneman graph appears more suitable.

## Conclusion

In this article we investigated the Most Parsimonious Phylogeny Estimation Problem from Single Nucleotide Polymorphism (SNP) haplotypes (MPPEP-SNP), a recent version of the phylogeny estimation problem that arises when input data consist of SNPs extracted from a given population of individuals. The MPPEP-SNP is NP-hard and this fact has justified the development of exact and approximate solution approaches such as those described in [1,19,22,26-28]. We explored the prospects for improving on the strategy of [1,2] using a novel problem formulation and a series of additional constraints to more precisely bound the solution space and accelerate implicit enumeration of possible optimal phylogenies. We present a formulation for the problem based on an adaptation of [23]’s mixed integer formulation for the Steiner tree problem extended with a number of preprocessing techniques and reduction rules to further decrease its size. We then show that it is possible to exploit the high symmetry inherent in most instances of the problem, through a series of strengthening valid inequalities, to eliminate redundant solutions and reduce the practical search space. We demonstrate through a series of empirical tests on real and artificial data that these novel insights into the symmetry of the problem often leads to significant reductions in the gap between the optimal solution and its non-integral linear programming bound relative to the prior art as well as often substantially faster processing of moderately hard problem instances. More generally, the work provides an indication of the conditions under which such an optimal enumeration approach is likely to be feasible, suggesting that these strategies are usable for relatively large numbers of taxa, although with stricter limits on numbers of variable sites. The work thus provides methodology suitable for provably optimal solution of some harder instances that resist all prior approaches. Our results may provide also useful guidance for strategies and prospects of similar optimization methods for other variants of phylogeny inference. In fact, if appropriately adapted, some of the results we presented here (e.g., symmetry breaking strategies) can be generalized with respect to other phylogenetic estimation criteria (e.g., the likelihood criterion) and provide important computational benefits.

## Competing interests

The authors declare that they have no competing interests.

## Authors’ contributions

The authors equally contributed to conceive the work and write the article. DC implemented the algorithms and performed computations. All authors read and approved the final manuscript.
